# Pragmatic Applications of RE-AIM for Health Care Initiatives in Community and Clinical Settings

**DOI:** 10.5888/pcd15.170271

**Published:** 2018-01-04

**Authors:** Russell E. Glasgow, Paul E. Estabrooks

**Affiliations:** 1 University of Colorado School of Medicine, Aurora, Colorado; 2 University of Nebraska Medical Center, Omaha, Nebraska

## Abstract

The RE-AIM (Reach Effectiveness Adoption Implementation Maintenance) planning and evaluation framework has been applied broadly, but users often have difficulty in applying the model because of data collection needs across multiple domains and sources. Questions in the more common “who, what, where, how, when, and why” format may be an effective guide to ensure that individual participants, organization staff, and the perspectives of the setting are considered in planning and evaluation. Such a format can also help users in typical community and clinical settings to identify which outcomes are most valued and to focus limited measurement resources. Translations of RE-AIM that are easy to understand and apply are needed for application in real-world community and clinical settings where research and evaluation resources are limited. The purpose of this article is to provide simplified, pragmatic, user-centered and stakeholder-centered recommendations to increase the use of RE-AIM in community and clinical settings and in translational research.

## Introduction

The RE-AIM (Reach Effectiveness Adoption Implementation Maintenance) framework was first developed to help make research findings more generalizable by encouraging scientists and evaluators to balance internal and external validity when developing and testing interventions (1). The goal was to produce programs and policies with a higher likelihood for uptake and sustainability in typical community or clinical settings. The constitutive definitions of the RE-AIM dimensions are straightforward and intuitively appealing for community and clinical organizations. Reach is the number, proportion of the intended audience, and the representativeness of participants compared with the intended audience. Effectiveness (or efficacy, depending on the design) is the degree to which the intervention changes health outcomes and quality of life, including producing unintended or negative results. Adoption is the number and proportion of settings and staff members that agree to initiate program or policy change and how representative they are of the intended audience in terms of the setting and the staff. Implementation is the degree to which those settings and staff members deliver a program or apply a policy as intended, the adaptations made, and the related costs. Finally, maintenance is sustained effectiveness at the participant level and sustained (or adapted) delivery at the setting or staff level (1).

The RE-AIM framework encourages planning strategies that can reach the most people who have health disparities, effectively achieve and maintain positive health outcomes, be widely adopted by diverse settings and staff, be consistently implemented at a reasonable cost, and be sustained among varied settings by staff members with a range of expertise (2). From an evaluative perspective, the added focus on representativeness, maintenance, and organizational level factors provides an opportunity to include, but also move beyond, considering efficacy or effectiveness as the only needed indicator of intervention success.

The Diabetes Health Connection, which included interactive technology and health coach support for patients with diabetes, provides an example of how RE-AIM can be applied to a programmatic intervention (3). The study documented a 38% participation rate and that participants were representative of the intended audience (reach). When compared with a health-risk appraisal intervention, the effect size was modest but significant and was positive for primary outcomes and quality of life changes (effectiveness). Twenty percent of the clinics approached and 79% of the staff of those clinics agreed to deliver the intervention, with larger HMO (health maintenance organization)-affiliated clinics being more likely to participate (adoption). The intervention was delivered consistently across participating staff members with an implementation rate of nearly 100%, at a cost of $547 per participant (implementation). Finally, maintenance of effects and organizational delivery of the intervention were beyond the scope of the trial but could have been assessed by examining metrics associated with effectiveness for 6 months or longer, postparticipant completion of the intervention (individual level maintenance), and setting-level maintenance based on the sustainability of implementation metrics.

Although RE-AIM has been applied for varied public health issues and intervention targets, challenges arise when considering the planning and evaluation of translational projects in typical clinical or community settings where interventions may be complex and multileveled. Furthermore, primary data collection across all RE-AIM indicators is challenging given available resources. For example, some users have reported that doing a “full RE-AIM application” is overly burdensome or not possible. In such cases, a pragmatic application of RE-AIM may be warranted (4). We feel that full use of RE-AIM and going through all of the questions for planning purposes is possible. However, it is often more challenging for people in community and clinical settings to report results on all RE-AIM dimensions without research funding.

Even with substantial funding, literature reviews on studies reporting use of the RE-AIM framework have generally produced only incomplete applications of the model. Kessler et al reviewed 42 National Institutes of Health (NIH) grant applications that used the RE-AIM framework and found that only 10% proposed to address all 5 RE-AIM dimensions (5). Grants proposed to address between 44% and 78% of the key criteria within a given dimension. In particular, data on the representativeness of staff members delivering programs were proposed only 31% of the time. Gaglio et al reviewed 71 recent publications, stating that they described use of the RE-AIM framework, and found that only 44 reported on all RE-AIM dimensions (6). Within RE-AIM dimensions, reporting was sparse for data on all elements with setting adoption (eg, percentage and representativeness, reasons for declining [0%]; implementation [2%]; and maintenance [2%]). These data confirm our observation that, if even these well-funded NIH grants and published research studies employed RE-AIM only partially and inconsistently, fewer, well-resourced community and clinical projects understandably have challenges doing so. Although reporting comprehensively on all RE-AIM dimensions can be challenging, the RE-AIM model is more intuitively appealing and easier to apply than many alternative evaluation and translation frameworks. For example, the Greenhalgh (7), the Consolidated Framework for Implementation Research (8), and PRECEDE-PROCEED models (9) all have considerably more components, criteria, and measures that may or may not be of direct interest to clinical and community organizations interested in evaluating the public health impact of their work.

## Purpose

The purpose of this article is to provide and discuss the use of a series of a familiar set of “who, what, when, where, how, and why” pragmatic questions based on the RE-AIM framework to guide the planning and evaluation of intervention strategies (program, policy, guideline) when evaluation resources are limited. We also provide decision points to guide how RE-AIM information can be pragmatically gathered and used in planning programs and evaluating relevant outcomes. Following this, and to make more concrete pragmatic uses of RE-AIM, we provide example of pragmatic uses of RE-AIM from the literature (4,10) for both planning and program evaluation.

We present the key pragmatic planning questions and measuring suggestions for stakeholders to consider for each RE-AIM dimension (Table). Users are encouraged to consider these questions throughout the phases of planning and implementation — although considering these questions during planning is likely most useful. During planning, users should consider which dimensions are most relevant and which they have the resources to measure well. Users are encouraged to use ongoing evaluation during delivery as a method to identify areas where adjustments may be necessary to achieve the desired outcomes. After program conclusion, follow-up interviews based on the planning questions can be used effectively to probe and better understand key findings.

**Table:** Key Translation and Pragmatic Questions to Consider in Addressing the RE-AIM (Reach Effectiveness Adoption Implementation Maintenance) Dimensions

RE-AIM Dimension	Key Pragmatic Questions to Consider and Answer^a^
**R**each	WHO is (was) intended to benefit and who actually participates or is exposed to the intervention? Measured by number and similarity of participants to your target group.
**E**ffectiveness	WHAT are (were) the most important benefits you are trying to achieve and what is (was) the likelihood of negative outcomes? Measured by change on key outcome(s) and consistency across subgroups.
**A**doption	WHERE is (was) the program or policy applied and WHO applied it? Measured by what settings and staff take up the intervention and which do not.
**I**mplementation	HOW consistently is (was) the program or policy delivered, HOW will it be (was it) adapted, HOW much will (did) it cost, and WHY will (did) the results come about?
**M**aintenance	WHEN will (was) the initiative become operational; how long will (was) it be sustained (setting level); and how long are the results sustained (individual level)? Measured by longevity of effects (individual level) and program sustainability (setting level).

a Terms in parentheses are phrased for postintervention evaluation. The basic questions are phrased for use in program or policy planning.

**Who is (was) intended to benefit and who actually participates or is exposed to the intervention? **This is reach at the level of patients, clients, or participants. It is important to consider not only how many persons participate out of those intended or targeted, but also the characteristics of those who take part compared with those who do not. Given the impact of health disparities, participation levels of various key underserved and vulnerable subgroups are especially important.

**What are (were) the most important benefits you are trying to achieve and what is (was) the likelihood of negative outcomes? **“What” refers to the effectiveness and individual-level maintenance components of RE-AIM at the patient, client, citizen, or participant level. Responses to this question include defining the key or desired outcomes and the impact on different subgroups (eg, men vs women; those at high risk vs those at low risk; those with high incomes or education vs those with low incomes or education). Additional outcomes include quality of life indicators and any negative effects or unintended consequences from the program.

**Where is (was) the program or policy applied (and who applied it)? **The issue of participation is also related to the settings level. Called *adoption* in the RE-AIM framework, participation is critical to understand the number and types of organizations, clinics, or agencies that initiate a program or policy. This participation involves considering the characteristics of the settings that are approached or targeted to participate compared with those that actually do. It also involves understanding barriers to and facilitators of adoption as well as how the program or policy fits with organizational priorities and existing workflow. These issues are also important at the suborganizational level, particularly in identifying which staff members try the new compared with those who are resistant or wait for others to attempt adoption first (11).

**How consistently is (was) the program or policy delivered, what adaptations to the original plans were made, and how much does (did) it cost? “**How” refers to the delivery or *implementation* of the intervention. How will the program or policy be delivered, managed, or enforced? How will you track adaptations or changes to the program in different settings, by different staff members, or over time? And how will you ensure that such changes do not reduce effectiveness?

How questions also include the organizational resources that are required and how much the policy or program costs to implement. Different organizations and decision-makers are concerned about different types of costs (eg, initial vs ongoing, fixed vs marginal, personnel vs infrastructure or equipment). Many organizations are concerned with short-term or medium-term return on investment, but this often means different things to different people (12). At minimum, it is important to report the time and staff resources required so that people in other settings can decide if they have the resources to adopt a program or policy.

**When will (did) the initiative become fully operational, how long do results last, and how long will (was) the initiative sustained? **“When” refers to organizational, setting, or staff level *maintenance*. This dimension can be operationalized as the sustainability of the policy or program and includes consideration of many factors, including but not limited to resource availability; alignment of the policy or program with organizational mission, objectives, and goals; and integration into job descriptions and performance evaluations. The timing of results is often important to decision-makers, and the end goal of most policies and programs is sustainability so that activities become institutionalized or normalized as part of the way of doing business (13).

**Why will (did) the results come about? “**Why” questions are concerned with the underlying reasons for intervention’s success or failure and apply across RE-AIM dimensions. Why did they come about? Often why questions are approached via interviews or other qualitative approaches, such as focus groups with users, and in combination with other measures and as ways to probe outcomes (eg, a program may have little impact because few people participate; positive effects may be restricted to a subset of users; a program can only be implemented successfully by a particular type of organization or staff person).

## Application and Examples

We provide examples of practical application of RE-AIM to issues of planning and outcome evaluation. The “translated,” less jargon-based RE-AIM questions above may be especially helpful in comparing 2 or more alternative programs or actions.

**Figure 1:**
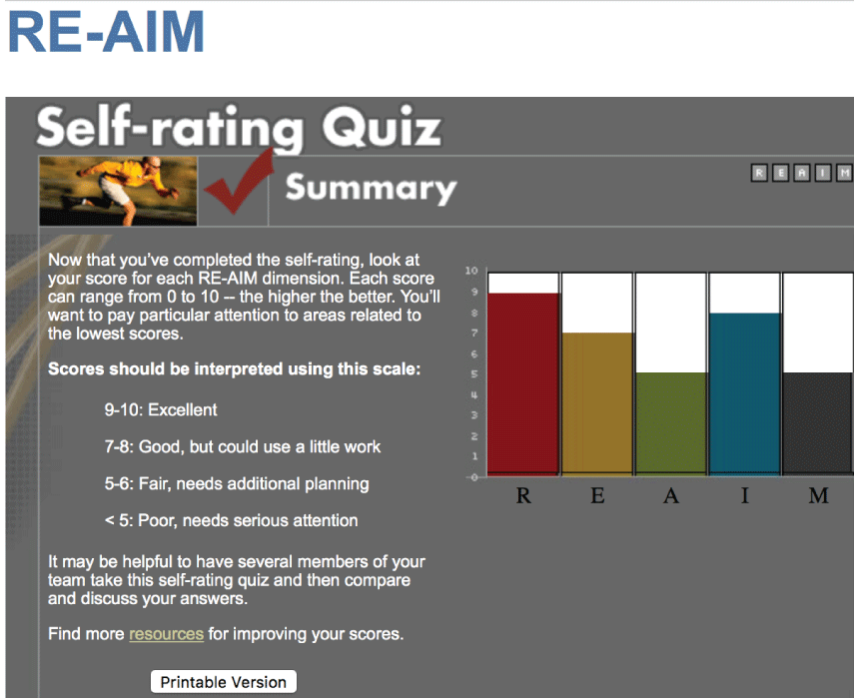
Summary of the RE-AIM Self-Rating Quiz with a scale for interpreting scores.

**Planning application.** Finlayson et al provide a helpful illustration of how RE-AIM can be used to help design a program, in their case, a program to prevent falls among people with multiple sclerosis (MS) (4). The International MS Falls Prevention Research Network used RE-AIM questions to structure initial discussions with clinicians, people with multiple sclerosis (MS), and representatives of professional and MS societies about the factors important to consider in the development of an MS falls-prevention program for application in multiple settings (who and where). They found a planning tool on the RE-AIM website to be helpful (http://re-aim.org/re-aim-as-a-planning-tool/). Figure 1 presents a prototypical example of the graphical summary of results from a stakeholder answering a series of RE-AIM program-planning questions similar to those presented here. Their group developed a series of practical questions to ask of a diverse group of people with MS and health professionals similar to the intended audience (who). Discussion of these questions, similar to the “who, what, where, how, when, and why” questions above about each RE-AIM dimension produced a series of 17 recommendations (what) for settings that were considering adopting falls prevention programs in local or regional MS societies (where) to use to plan for success. They found that using the RE-AIM framework early in their work helped to develop a feasible intervention that could be widely adopted and well implemented and a protocol that maximized the ability to translate research into practice. Because the Finlayson group’s application of RE-AIM was for planning purposes, they did not have data on results (effectiveness), maintenance (sustainability), or program costs (implementation). Their use of the RE-AIM self-rating quiz on the RE-AIM website (www.re-aim.org) provided initial estimates on most of these issues, but “why” interviews could have provided additional information for planning. A score from 1 through 10 is provided for each dimension, based on the extent to which the various issues within that dimension are addressed (Figure 1).

**Evaluation example.** Burke et al used the RE-AIM framework to evaluate an academic–community partnership approach to address childhood obesity (10). They identified children aged 8 to 14 years with a body mass index percentile ranking in the obese range as those intended to participate and benefit in the Children’s Health and Activity Modification Program (CHAMP). They also identified representativeness as the key participation or reach (who) indicator for their feasibility study and documented that the sample was representative of race, household income, and employment status when compared with the small regional city where the study was conducted — while also reporting on the number of participants and percentage of the total population that participated (10).

CHAMP was delivered at both university and community (YMCA) locations (ie, Where was the program applied?) and included staff from both organizations (10). Twenty-six delivery personnel participated, all with different levels of expertise but with experience in providing activities or events for children from the intended audience. CHAMP focused on weight for child participants and demonstrated significant weight reduction that was maintained for 6 months after the intervention was complete (ie, What was the benefit and likelihood of maintenance?). The question of when the initiative became fully operational and the duration of sustainability was not completely answerable in the CHAMP project; however, the study documented that of the 26 personnel who were involved in the initial implementation, all agreed to a second implementation, and additional staff were added based on participant feedback (10).

Implementation (How) of the CHAMP intervention strategies was more than 90%, and assessment of parent comprehension of intervention objectives was used as an indirect assessment of receipt of intervention components directed at the home (66% of content correctly identified) (ie, How was the intervention delivered?). Burke and colleagues also provided information on the cost of implementation across personnel, supplies, and dissemination with a total of cost of approximately $140,000 across 2 years (ie, How much did it cost?). Finally, qualitative feedback from participants indicated that the children found the program engaging and fun to participate in (Why). Participants also indicated that providing fun exercise experiences helped distract the children from the intensity of the activities; showing that a variety of healthy food options helped improve dietary intake; and engagement in the program helped the children feel better about themselves (all why questions related to program success) (10).

## Discussion

Although RE-AIM is less complex and more intuitive and understandable than many evaluation models and systems (6,14), it is still not easily applied in its full form by community and research teams (5,15). This article provides a common-sense presentation of how RE-AIM can be used to address “who, what, where, how, when, and why” questions important to decision-makers and how it can be used to plan and evaluate policies and programs. Practical application of selected RE-AIM dimensions can be done by specifying in advance what impact and outcomes are most important and by providing a rationale for the dimensions that are and are not addressed. Such reporting ensures that the pragmatic application and use of RE-AIM is justified and not just due to overlooking key issues (16).

These challenges in applying dissemination and evaluation models in applied or even research settings are not unusual or specific to RE-AIM. In many ways, this use of RE-AIM for decision-making is parallel to environmental impact assessment procedures or community-informed system dynamics planning (17) in that it can help ensure that many aspects and different effects, including potential negative and unintended consequences, are considered.

**Figure 2:**
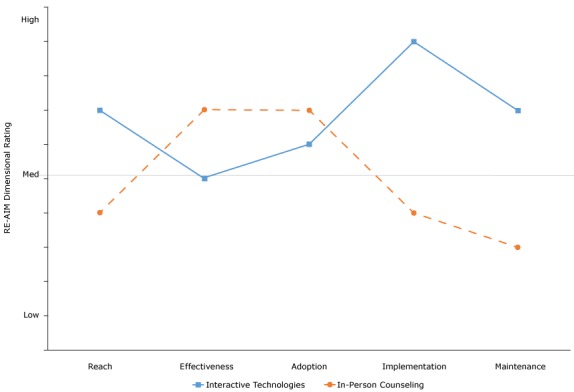
Results of comparison of application of RE-AIM questions to 2 programs. A score of 1 to <5 indicates low application, a score of 5 indicates medium application, and a score of >5 to 10 indicates high application. Abbreviation: RE-AIM, Reach Effectiveness Adoption Implementation Maintenance. **Table:** 

This practical application or translation of RE-AIM can be used to evaluate different approaches to a given problem or issue and for various programs, policies, and content areas. Figure 2 provides a summary example of the application of RE-AIM questions to 2 different programs that compare their results (projected or actual). This figure addresses the types of issues that environmental planners, community policy-makers, public health officials, health care decision-makers, and comparative-effectiveness researchers have to make.

RE-AIM Component	Interactive Technologies	In-Person Counseling
	RE-AIM Dimensional Rating	
Reach	7	4
Effectiveness	5	7
Adoption	6	7
Implementation	9	4
Maintenance	7	3

In summary, the use of who, what, where, how, when, and why questions based on the RE-AIM model should be useful in both community and clinical settings with limited resources. All of these questions and the RE-AIM framework and tools have been placed in the public domain (www.re-aim.org). We encourage their use and hope that in the future there will be sufficient reports of use of this approach to enable summarizing of results and lessons learned from such applications.
